# Development of a Compartmental Pharmacokinetic Model for Molecular Radiotherapy with ^131^I-CLR1404

**DOI:** 10.3390/pharmaceutics13091497

**Published:** 2021-09-17

**Authors:** Sara Neira, Araceli Gago-Arias, Isabel Gónzalez-Crespo, Jacobo Guiu-Souto, Juan Pardo-Montero

**Affiliations:** 1 Group of Medical Physics and Biomathematics, Instituto de Investigación Sanitaria de Santiago (IDIS), 15706 Santiago de Compostela, Spain; sara.neira.castro@hotmail.com (S.N.); maria.araceli.gago.arias@sergas.es (A.G.-A.); isabel.gonzalez.crespo@sergas.es (I.G.-C.); 2 Department of Medical Physics, Complexo Hospitalario Universitario de Santiago de Compostela, 15706 Santiago de Compostela, Spain; 3 Institute of Physics, Pontificia Universidad Católica de Chile, Santiago de Chile 7820436, Chile; 4 Department of Medical Physics, Centro Oncolóxico de Galicia, 15009 A Coruña, Spain; jacobo.guiu@cog.es

**Keywords:** biokinetics, compartmental model, iodine, molecular radiotherapy, CLR1404

## Abstract

Pharmacokinetic modeling of the radiopharmaceuticals used in molecular radiotherapy is an important step towards accurate radiation dosimetry of such therapies. In this paper, we present a pharmacokinetic model for CLR1404, a phospholipid ether analog that, labeled with ^124^I/^131^I, has emerged as a promising theranostic agent. We follow a systematic approach for the model construction based on a decoupling process applied to previously published experimental data, and using the goodness-of-fit, Sobol’s sensitivity analysis, and the Akaike Information Criterion to construct the optimal form of the model, investigate potential simplifications, and study factor prioritization. This methodology was applied to previously published experimental human time-activity curves for 9 organs. The resulting model consists of 17 compartments involved in the CLR1404 metabolism. Activity dynamics in most tissues are well described by a blood contribution plus a two-compartment system, describing *fast* and *slow* uptakes. The model can fit both clinical and pre-clinical kinetic data of ^124^I/^131^I. In addition, we have investigated how simple fits (exponential and biexponential) differ from the complete model. Such fits, despite providing a less accurate description of time-activity curves, may be a viable alternative when limited data is available in a practical case.

## 1. Introduction

The phospholipid ether analog CLR1404 (18-(p-iodophenyl)octadecyl phosphocholine) has emerged in the last years as an interesting compound for nuclear medicine applications. The derived radiopharmaceuticals ^124^I- and ^131^I-CLR1404 are of potential interest in molecular radiotherapy (MRT) applications as a theranostic duo. Several preclinical studies have demonstrated tumor growth suppression, high tumor and metastasis specificity, prolonged retention for a variety of cancer cell lines, and low healthy tissue toxicity [1,2,3,4,5,6]. Studies have already been conducted in human patients [7,8,9,10], and the drug is currently at the Phase II stage of clinical development [11]. The administration of ^124^I-CLR1404 allows for a pre-treatment evaluation of the patient’s biokinetics by PET imaging, which offers higher image quality than SPECT, while ^131^I-labeling provides a therapeutic effect due to its longer half-life and decay scheme.

Biokinetic compartmental models have been long employed for internal dosimetry calculations in nuclear medicine. These mathematical tools allow modeling the metabolic mechanisms of an administered radiopharmaceutical and to characterize its activity distribution in the body over time. Therefore, they are an important tool for internal dosimetry in MRT. Historically, they have been employed for deriving average activity coefficients based on activity measurements from a patient cohort [12]. These tabulated values serve as the basis for the well-established MIRD formalism, a dosimetric methodology based on the use of non-individualized dose-activity coefficients that is widespread both in therapy and diagnostic applications [13]. In this regard, several biokinetic models of radiopharmaceuticals have been developed, both for therapy and radiation protection applications [14,15,16,17,18,19,20].

During the past few years, there has been a shift in the guidelines towards dosimetry calculations. Currently, the legislation of some countries mandates personalized internal dosimetry for each patient that undergoes a procedure involving radiopharmaceuticals, such is the case of the European Council Directive 2013/59/Euratom [21] in the European Union. Accurate internal dosimetry methods for these procedures could allow individual treatment optimization, risk estimation associated with radiation exposure, and evaluation of the treatment outcome [22]. One of the main requisites for achieving accurate radiation dose estimations is the careful characterization of the biokinetics, as it represents the major source of uncertainty of non-individualized calculations [23]. Nowadays, many of the proposed methods and platforms for individualized dose estimations rely on patient-specific biokinetic experimental data, such as PET/SPECT images acquired at different time-point after administration [24,25,26,27,28,29], which are then fitted to a certain functional form to obtain the individualized activity distribution per patient.

The characterization of the radiopharmaceutical uptake is possible if enough dynamic or sequential images are acquired over time. Nonetheless, exhaustive data acquisition is not always a possibility in clinical practice, where the most common scenario is that only a few images are available. In this context, a biokinetic model can be employed as the functional form needed to obtain realistic and biologically coherent activity distributions in individual patients, fitting the model to available experimental data in order to *fill the gaps* and obtain individualized time-activity curves. Biokinetic models can also be used to study and optimize the acquisition time-points in a clinical scenario.

In this work, we present the development of a compartmental biokinetic model for ^124^I/^131^I-CLR1404. The model was built upon previously available experimental data [7,8] by fixing a forcing function for the blood and decoupling each data series into compartmental sub-systems, and then using a rigorous methodology including the goodness-of-fit, Sobol’s sensitivity analysis, and the Akaike Information Criterion to construct the optimal form of the model. It aims at providing a comprehensive interpretation of the metabolic behavior at the tissue level of this novel radiopharmaceutical and assisting in the individualized dosimetry of MRT patients. The potential of the model as a dosimetric tool was tested, together with other simpler alternatives that might be suitable for individualized dosimetry calculations of ^124^I/^131^I-CLR1404 applications. To the best of our knowledge, this is the first compartmental biokinetic model that has been proposed for ^124^I/^131^I-CLR1404, and it may prove useful to analyze upcoming clinical data for these radiopharmaceuticals.

## 2. Materials and Methods

### 2.1. Clinical Data and Data Processing

As ^124/131^I-CLR1404 is a novel radiopharmaceutical, not yet approved for clinical use nor commercially available, we have relied on limited data available in the literature. We imported the data from the study by Besemer et al. [7], where a breast-cancer patient was administered pre-treatment and therapeutic activities of ^124^I- and ^131^I-CLR1404, respectively. In that study, the activity concentration at different times was measured with PET (^124^I) and SPECT (^131^I) and reported for the liver, lungs, bone marrow (BM), heart wall, spleen, kidneys, spinal cord, and tumor (figure 2 from [7]). We also relied on blood and urine activity data from [8], as such data were not reported by Besemer et al. In the latter study [8], the biokinetics of the ^131^I-CLR1404 was examined, and both blood and urine samples were collected over time for a patient cohort.

The imported data from the mentioned publications were processed to adapt them to the requirements of our study. The activity concentrations per organ mass that were reported in [7] were converted into absolute activity concentrations. Because the specific organ masses of the patient were not published, the female reference mass values were adopted from the ICRP publications [30,31], as shown in Table 1. Activities in the spinal cord, which are reported in [7], were not considered for the development of the model due to its low activity uptake. For the organs, both the SPECT and PET data sets were included in the analysis: PET data extending up to 120 h post-injection, and SPECT data extending from 120 h to 505 h (the first SPECT data-point was in the range covered by the PET and therefore was discarded). In the case of the tumor, there was a considerable mismatch between the PET and SPECT activity concentration. Therefore, the SPECT points were fit to an exponential function and scaled to match the last PET time-point (120 h). For data fitting, 5% relative uncertainties were considered for each experimental point, except for the SPECT-derived tumor data points, where a 10% value was taken instead due to the discrepancy mentioned above.

The data from [8] were processed to include physical decay. Mean values and standard deviations in the studied cohort were taken for both urine and blood activity measurements. One data point for urine (t=120 h) was omitted from the analysis since its dispersion was too low to faithfully represent the cohort distribution.

### 2.2. Experimental Data in Xenograft Models and Data Processing

For further validation of the model, we employed data available from a study in mice. Baiu et al. [1] inoculated a diagnostic activity of ^124^I-CLR1404 to xenograft mouse models with different tumor cell lines. The activity evolution in tissue was studied by acquiring several PET/CT scans over time, and the resulting activities were extrapolated to ^131^I-CLR1404 by correcting for the physical decay of ^131^I. We imported and processed the results of these measurements, reported in their supplemental tables 2–5, to fit the proposed model. However, unlike in data reported in [7], no excretion or blood activity data were available.

In that publication, organ activities were expressed as concentrations, in units of %/g, although the corresponding mice organ masses were not published. To transform the activity concentrations into absolute activities, we assumed the organ masses for a 30 g murine phantom model reported by [32], showed in Table 2. The mass of the organs was assumed to be constant during the time interval between the injection and the complete elimination of the radiopharmaceutical from the body.

After the diagnostic injection, Baiu et al. administered a therapeutic injection of ^131^I-CLR1404 and measured the volume evolution of the tumor with time (figure 4 in [1]). This change in volume was taken into account when calculating activities in the tumor.

**Table 2:** Employed organ masses for scaling activity concentrations [%/g] into absolute activities [%], and theoretical blood fractions. Masses and blood fractions were taken from [32,33], except for: ^a^ mass from [34], and ^b^ same blood fraction as for the humans, taken from [30].

Organ	Mass [g]	Initial Blood Fractions [%]
Heart ^b^	0.291	1.0
Kidneys (sum)	0.374	4.8
Liver	2.150	23.0
Lungs (sum)	0.107	2.6
Marrow ^a,b^	1.049	4.0

### 2.3. Mathematical Methods

Best-fits of the biokinetic model to experimental data were obtained with an in-house optimization code based on a simulated annealing algorithm (SA) [35], which was implemented in MATLAB (The MathWorks, Natick, MA, USA). The objective function was formulated as a weighted residual sum of squares, WRSS:

(1) $$\begin{array}{c} \mathrm{WRSS}=\sum_{s=1}^{S}\sum_{p=1}^{P_{S}}\frac{{\left(A_{s,p}^{\mathrm{obs}}-A_{s,p}^{\mathrm{th}}\right)}^{2}}{\sigma_{s,p}^{2}}, \end{array}$$

where $A^{\mathrm{obs}}$ is the measured relative activity and $A^{\mathrm{th}}$ is the model prediction, *s* runs over the number of organs/tissues, and *p* runs over the number of time-points per organ/tissue. The weights were defined as the inverse of the measurement variance, $\sigma_{s,p}^{2}$.

As the experimental activity in each organ/tissue will be modeled as a blood fraction plus one or several *dry tissue* contributions, modeled activities are calculated as:

(2) $$A_{s}^{\mathrm{th}}\left(t\right)=\sum_{i\in s}y_{i}\left(t\right)+f_{s}\;y_{\mathrm{blood}}\left(t\right),$$

where $A_{s}^{\mathrm{th}}\left(t\right)$ is the activity in the organ/tissue *s* at time *t*, $f_{s}$ is its blood fraction, and *i* represents each one of the compartments that make up the corresponding organ/tissue *s*. The reference blood fractions are reported in Table 1 and were extracted from [30] (Table 7.7.2 in that reference). Both the urinary bladder and tumor blood fractions were approximated to be negligible. Best-fitting blood fraction values were constrained during the optimization to a range of ±30% of their reference value.

We have used the Akaike Information Criterion (AIC) with sample size correction [36] to compare models with different numbers of parameters at several steps of this work. This methodology ranks models according to the value of the objective function and number of parameters:

(3) $$AIC_{c}=N\log\left(\frac{\mathrm{WRSS}}{N}\right)+2K+\frac{2K(K+1)}{N-K-1},$$

where *N* is the total number of experimental data points and *K* the number of model parameters. Models with lower ${\mathrm{AIC}}_{c}$ are preferred.

Sensitivity analysis was also used to search for potential sources of improvement and simplification during the development of the biokinetic model. We relied on the methodology of Sobol indices [37,38] to investigate which parameters affected the fitting of experimental data the most and whether some of them could be discarded. First- and total-order Sobol indices come from the decomposition of the variance of a function output as a function of the variance of its parameters. The first-order indices, $S_{i}$, measure the impact of the variation of each parameter, being $S_{i}∼1$ an indicative of a highly influential parameter, and $S_{i}∼0$ hinting that the parameter under consideration has no direct effect on the output. On the other hand, the total-order indices ($S_{T,i}$) consider not only the variation of each parameter separately but also of all the different interactions between them. That is, a parameter can affect the output when it is varied in combination with others. A low value of the total-order index ($S_{T,i}∼0$) is an indicator that the parameter is not relevant (either individually or in association with others) and therefore it can be omitted from the model. Details about the Sobol methodology are provided in the Supplementary Materials.

### 2.4. Biokinetic Model Construction: Decoupling and Forcing Function

The biokinetic model was built by following a systematic approach based on a decoupling process applied to experimental data and using the goodness-of-fit, Sobol’s sensitivity analysis, and the Akaike Information Criterion. This methodology was used on the clinical data reported in Section 2.1. Pre-clinical data (Section 2.2) were used for further validation, but not during model construction.

The system was decoupled in subsystems by employing a forcing function linked to the blood compartment, as described in [39]. This methodology also allowed to obtain an approximate initial value for the rate constants which was employed during the final fitting. This process was performed as follows: first, a forcing function was defined by fitting the blood data to a biexponential function; then, this functional shape was used to lock the blood activity evolution of the subsystem; finally, this model structure was fitted to the experimental data of the organ under study and its suitability evaluated. Different alternatives for the functional modeling of activities in an organ/tissue were investigated.

**Figure 1:**
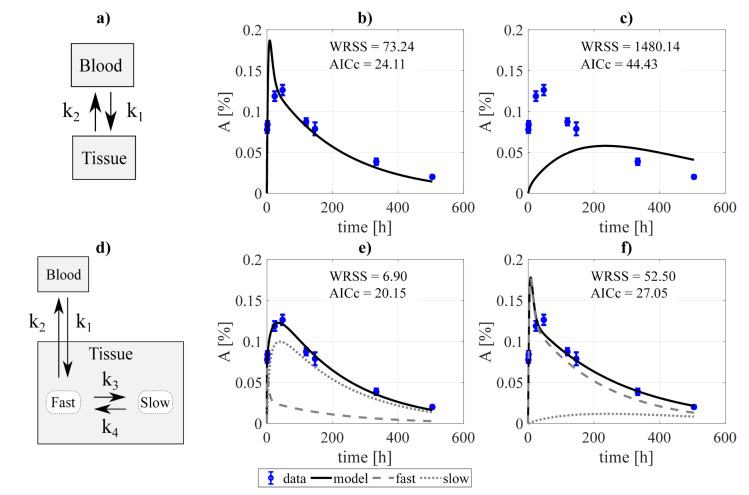
Example of the decoupling method for the tumor subsystem, constrained by a biexponential forcing function for the blood compartment. Panel (**a**) shows a simple two-compartment model which was fitted to the data assuming: (**b**) bidirectional blood-tissue exchange, and (**c**) only one direction blood-tissue ($k_{2}$ = 0). Panel (**d**) shows a three-compartment model which was fitted assuming: (**e**) four exchange directions blood-fast and fast-slow, and (**f**) three exchange routes (slow-fast is canceled, $k_{4}$ = 0). WRSS and ${\mathrm{AIC}}_{c}$ refer to the weighted residual sum of squares and Akaike Information Criterion with sample size correction of the fits.

**Figure 2:**
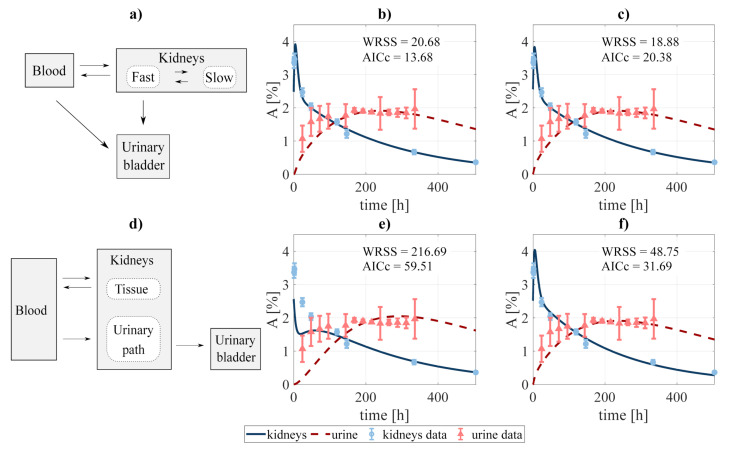
Compartmental design to perform the system decoupling for the excreted activity data: (**a**) the activity to be excreted can come from the fast component of the kidneys and directly from the blood. The data was fit to several combinations of the system, defined by removing none or some rate constants, among them: (**b**) blood-bladder and slow-to-fast components removed, and (**c**) none were removed. A second scheme (**d**) was tested, where the excreted activity travels from the blood to the urinary bladder passing through the urinary path (structure analog to that used by [40] for FCH). The fitting was carried out for the cases where (**e**) tissue-blood component, and (**f**) no components were removed. WRSS and ${\mathrm{AIC}}_{c}$ refer to the weighted residual sum of squares and Akaike Information Criterion with sample size correction of the fits.

This procedure is illustrated in Figure 1 and Figure 2. Mathematically, the equation describing the simplest alternative (see Figure 1a) is given by:

(4) $$\begin{array}{c} \frac{dy_{\mathrm{tissue}}}{dt}\left(t\right)=k_{2}\;h_{\mathrm{blood}}\left(t\right)-k_{1}\;y_{\mathrm{tissue}}\left(t\right), \end{array}$$

where $h_{\mathrm{blood}}$ is the biexponential forcing function, $y_{\mathrm{tissue}}$ is the time-activity curve in the tissue, and $k_{1}$ and $k_{2}$ are the rate constants that communicate the compartments. The resulting equations were fitted to the experimental data of the organ under study by considering that the measured activity comprised two components: a fraction due to the blood content of the organ plus the contribution of the tissue compartments, which represent the proportion of *dry tissue* (meaning tissue without blood). As a consequence, the number of parameters of the model is the number of *k*-rate constants plus the blood fractions. The precise details regarding the fitting process were provided in the previous section.

### 2.5. Model Simplifications and Clinical Application

Usually, in a clinical scenario, only a few measurements can be performed. Complex biokinetic models may be limited in this situation because the restricted set of experimental data may not allow to uniquely determine model parameters. In such a scenario, simple models may prove more useful. Here, we have characterized the performance of three simplifications compared to the full biokinetic model, and evaluated the trade-off between the prediction error and the number of required measurements:

(i) a simplified version of the full biokinetic model with only one compartment per organ (two exchange rates), except for the tumor.
(ii) a biexponential fit, both to the whole experimental dataset and to different combinations of four experimental time points.
(iii) a monoexponential fit (physical decay) to only one of the experimental data points (24 h, 48 h, or 120 h).

We compared the performance of the different approximations in terms of integrated activities for each organ/tissue.

## 3. Results

### 3.1. Biokinetic Model Construction

The procedure of model construction through decoupling is illustrated in Figure 1 (for the tumor). Two structures were proposed for each organ/tissue. The simplest model structure considered the tissue to be described by only one compartment, Figure 1a. The experimental activity curve was fitted considering a bi-directional exchange between tissue and blood ($k_{1}$≠ 0, $k_{2}$≠ 0, Figure 1b), or the tissue as a sink ($k_{2}$ = 0, Figure 1c). A second model structure was studied, with the tissue conformed by two compartments, *fast* and *slow* (Figure 1d). Analogously to the previous case, the data was fit considering four exchange directions ($k_{1}$≠ 0, $k_{2}$≠ 0, $k_{3}$≠ 0, $k_{4}$≠ 0, Figure 1e), and the slow component to behave as a sink ($k_{4}=0$, Figure 1f). The resulting fits were evaluated according to the value of the objective function and the ${\mathrm{AIC}}_{c}$, from which the structure to be incorporated into the final model was selected. For the example presented, the chosen model was the two-compartment and four rate constants model (Figure 1e).

In addition, two slightly different scenarios were investigated for the particular case of modeling the excreted activity. In the first one (Figure 2a), the activity in the urinary bladder can come both from the fast component of the kidneys and directly from the blood. In the second scenario (Figure 2d), the activity in the urinary bladder travels from the blood to the bladder passing through the *urinary path*. The latter scenario is analog to that implemented by [40] for modeling the biokinetics of fluorocholine. As in the previous case, different fits were performed involving different combinations of the rate constants. Finally, the process led to choose the structure shown in Figure 2a, excluding the blood-bladder path (fit shwon in Figure 2b), as the best option, with an ${\mathrm{AIC}}_{c}$ of 13.68 (versus 20.38, 31.69, and 59.51 for the alternatives shown in Figure 2c,e,f, respectively).

**Figure 3:**
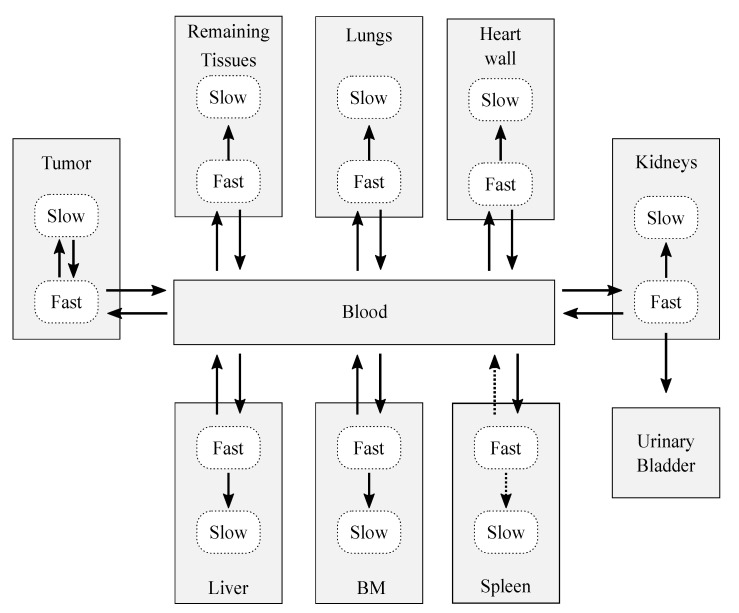
Sketch of the biokinetic model proposed for CLR1404. The dotted arrows represent those contributions that were initially considered after the decoupling analysis, but which were later discarded after the data fitting and sensitivity analysis. The solid arrows represent the exchange paths of the final model.

After the decoupling process, the resulting complete model (Figure 3) was conformed by 18 compartments, which represent 10 structures: 7 organs (the spleen, bone marrow, heart wall, kidneys, liver, and lungs), a remaining tissues structure (accounting for the non-explicitly-modeled tissues), the tumor, blood, and urinary bladder contents. The decoupling analysis revealed that, in general, each organ was well modeled with two compartments and four adjustable parameters: three rate constants that describe the flow of CLR1404 between fast-slow compartments and the blood pool, and an extra parameter to account for the blood volume that is permanently held by each tissue, the *blood fraction*. Therefore, each organ compartment that was characterized in the model is interpreted as *dry tissue* (no blood fraction included).

The compartmental model presented in Figure 3 is mathematically described by the system of ordinary differential equations (ODE):

(5) $$\frac{dy_{i}}{dt}\left(t\right)=\sum_{j}k_{j,i}\;y_{j}\left(t\right)-\sum_{j}k_{i,j}\;y_{i}\left(t\right)-\lambda \;y_{i}\left(t\right),$$

where $y_{i}$ is the percentage of initial injected activity in the *i*-th compartment, $k_{i,j}$ is the rate constant from compartment *i* to compartment *j*, and λ is the physical decay constant of the radioisotope. The detailed ODE system is presented in the Supplementary Materials.

### 3.2. Model Refinement: Data Fitting

This first model proposal (Figure 3) was analyzed in search of sources of refinement. Firstly, the forcing function restriction for the blood was lifted and the complete model was fitted to the available experimental data. The fitting process was performed by minimizing the WRSS given by Equation (1), with activities given by Equation (2).

Data-fitting showed that the fast-to-slow rate constant for the spleen converged to zero. Therefore, the model for the spleen was simplified to include just one compartment.

### 3.3. Model Refinement: Sobol Analysis

The first and total-order sensitivity indices were calculated for each model parameter. The variance analysis was performed through a Monte Carlo calculation where the model parameters were perturbed following a normal distribution of zero mean and relative standard deviation of 10%. It involved a total computational cost of M(K+2) runs, with $M=5\times 10^{4}$ being the number of samples for each parameter, and *K* the number of model parameters.

The Sobol analysis, whose results are shown in the Supplementary Materials file (Tables SM1 and SM2), led to an additional simplification of the model: the spleen-to-blood exchange was removed (and therefore the spleen behaves as a pure sink) since the first and total-order Sobol indices were null.

### 3.4. Final Model Fitting

The final biokinetic model consisted of 17 compartments involving 24 rate constants and 6 blood fractions, as shown in Figure 3 (solid arrows). The model was classified as globally identifiable by the software tool DAISY [41], setting initial conditions of null initial activity in all compartments except for the bolus injection in the blood.

**Figure 4:**
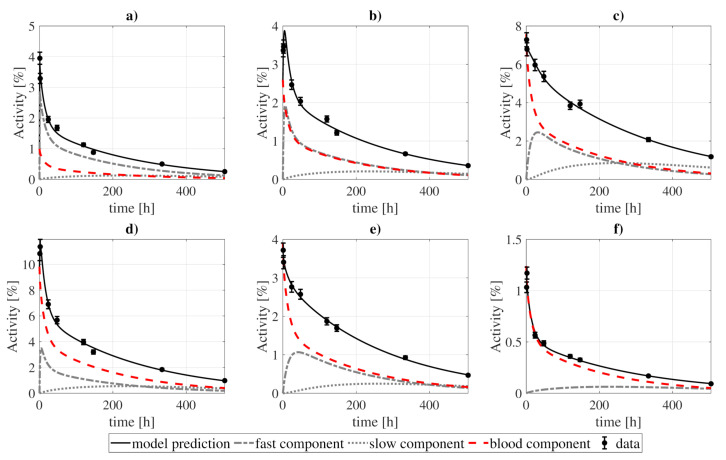
Model fitting to ^131^I-CLR1404 biokinetic data for several organs: (**a**) heart wall, (**b**) kidneys, (**c**) lungs, (**d**) liver, (**e**) BM, and (**f**) spleen. The contribution of each compartment (fast, slow, and blood) to organ activities is represented by the dashed lines, while the solid lines show the sum of those contributions. The presented standard deviations were artificially estimated as a 5% of the measurement, as no information regarding uncertainties was reported in the studies of reference. Experimental and modeled activities take into account the physical decay of the ^131^I.

**Figure 5:**
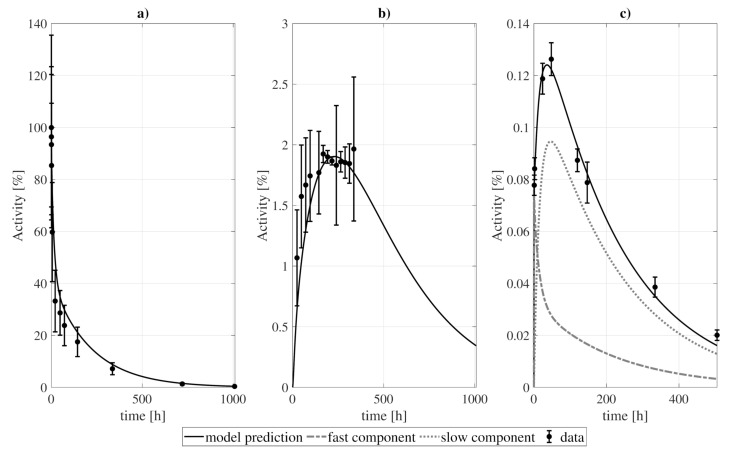
Model fitting to (**a**) blood, (**b**) urine, and (**c**) tumor data. For the tumor, a standard deviation of 5% of the PET experimental measurements and 10% for the SPECT ones (last 3 points) were taken. For blood and urine, the standard deviation represents the dispersion of the patient group of [8]. Experimental and modeled activities take into account the physical decay of the ^131^I.

In Figure 4 and Figure 5 we present the model fit to the experimental data, while the best-fitting values of the model parameters are shown in Table 3. The goodness-of-fit, computed as the WRSS divided by the number of degrees of freedom, reached a value of 1.22. The uncertainties of the best-fitting model parameters were estimated from a Monte Carlo simulation [42], where the model was fitted to different sets of randomly perturbed experimental measurements (by using a normal distribution with the standard deviation of the data). This process was repeated for 100 runs, after which the uncertainty was estimated from the standard deviation of the best-fitting parameters.

Although the structure presented in Figure 3 was the best alternative, several tests were performed for several modified versions during the construction of the model, the metrics of which are presented in Table 4.

### 3.5. Comparison with Simpler Models

**Figure 6:**
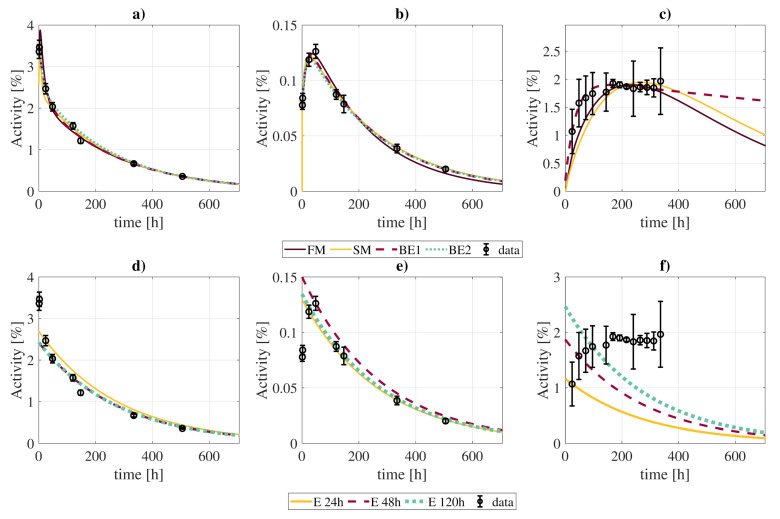
Comparison of best fits of time activity curves (kidneys, panels (**a**,**d**); tumor, panels (**b**,**e**); excreted activity, panels (**c**,**f**)) obtained with the full model and different approximations: “FM” (full model); “SM” (simplification of the full model without slow components except for RT and tumor); “BE1” (biexponential fit with the entire dataset); “BE2” (biexponential fit with only 4 points at [2, 24, 120, 505] h); “E 24 h”, “E 48 h”, “E 120 h” (exponential fits considering the physical decay of activities measured 24 h, 48 h, and 120 post-injection, respectively).

The simpler biokinetic models were compared to the full biokinetic model in terms of cumulated activity: the results are shown in Figure 6 and Table 5. For the simplified biokinetic model, the prediction of the cumulated activity differed less than 5% for most cases, except for the tumor and urinary bladder contents, with differences up to 8% and 12% respectively. However, despite the qualitative agreement on cumulated activities, the goodness-of-fit increased with the simplification up to 1.91. The computation of the ${\mathrm{AIC}}_{c}$ also discarded this version of the model as the best option, with a value of 93.40 versus 75.76 for the full model (Table 4).

The biexponential fits showed generally good agreement with the full biokinetic model in terms of the cumulated activities, with differences in the [−1,6]% range, except for the contents of the urinary bladder, where the clearance rate of the biexponential fit is much slower, resulting in larger integrated activities. If the biexponential fit is limited to four time-points, best results are obtained for measurements taken at [2, 24, 120, 505] h (Supplementary Materials, Figure SM1).

The exponential fits to a single data point (at *t* = 24 h) showed much larger disagreements, with differences between [−75,24]%. However, such differences were lower when using late time-points (48 and 120 h). In this case, cumulated activities estimates did not suffer from large deviations, except for the tumor (11%), lungs (−13%), and urinary bladder contents (−46%).

### 3.6. Preclinical Data Fitting

**Figure 7:**
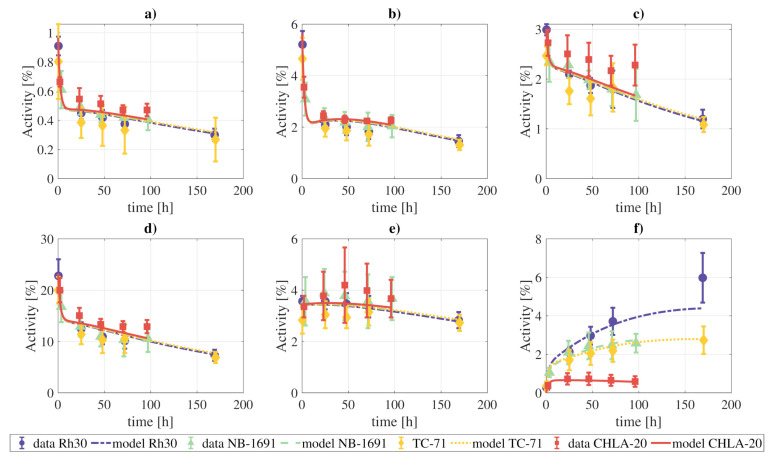
Fitting of the complete model to the combined tissue activities reported for xenografts in [1]: (**a**) lungs, (**b**) heart, (**c**) kidneys, (**d**) liver, (**e**) marrow, (**f**) tumor. The parameters which govern the tumor compartments were allowed to vary individually for each tumor cell line. The objective function reached a value of WRSS=58.02.

The activity data from the xenograft models were also fit to the final model. We simultaneously fit all the measurements of the four tumor cell lines, meaning that the rate constants and blood fractions are the same for the four tumor lines, except for parameters that govern the behavior of the tumor, which were allowed to vary independently for each one of the tumor lines. The results of the fitting are presented in Figure 7, and the model can provide a qualitatively good fit of the experimental data.

If the constraint on the rate constants and blood fractions indicated above is lifted, the fits are qualitatively better, due to the higher number of degrees of freedom (Supplementary Materials, Figures SM2, SM3, SM4, and SM5).

## 4. Discussion

In this work, we followed a rigorous methodology for the progressive construction of a biokinetic model for CLR1404 by using the decoupling methodology, with refinements arising from the fitting results and the sensitivity analysis. In this model, the activity contained within an organ/tissue is interpreted as a sum of several components. On the one hand, the blood fraction in each organ/tissue, being its relative contribution to the total integrated activity important in most cases (varying from a minimum of 21% in the heart wall to a maximum of 77% in the spleen). On the other hand, the organ’s *dry tissue* is modeled by two components: a *fast* one, which dominates at early instants post-injection but loses weight over time, when the *slow* component becomes more important. This model structure provided best-fits for the majority of organs, except for the spleen, where the activity exchange mechanism is simpler: the resulting optimization and sensitivity analysis showed that the spleen could be well approximated by a single component, with a sink-like behavior due to the slow return to the blood. The tumor also presents a particular biokinetics: our methodology does not show the need for permanent uptake (sink) of activity in the tumor tissue (Figure 1). However, this behavior should be further experimentally tested in other patients and tumor lines.

Regarding excretion, different approaches were investigated, as shown in Figure 2. The analysis showed that the best alternative was that represented in Figure 2b, where the paths blood-to-urine and kidneys slow-to-fast were neglected (although taking those paths into account improves the fit, this is not favored by the ${\mathrm{AIC}}_{c}$). Nonetheless, differences between different excretion models were not critical, which opens the door for a re-evaluation of the chosen scheme for a different data set.

When developing the model, it was observed that in some cases organ data could be described by very simple structures, for instance, by using a single compartment for the liver or the heart, as shown in Table 4. However, when performing statistical tests, the complete model was always prioritized by the WRSS and the ${\mathrm{AIC}}_{c}$, although the difference was modest.

This compartmental model can describe the biokinetics of ^124^I/^131^I-CLR1404, as shown by the goodness-of-fit, and represents the best trade-off between the number of parameters and goodness-of-fit. However, although for the available experimental data the presented model showed the best results, it might suffer from modifications if analyzed throughout a more coherent dataset. The limited set of experimental data, their heterogeneity (the available experimental data consisted of a conglomerate of measurements from different sources), and the fact that they were adapted from the literature, is certainly a limitation of the present study. The performance of more exhaustive experiments, for a larger cohort of patients and specifically targeting the model development, may result in a better understanding of metabolism and, consequently, a more reliable model. Accordingly, the resulting cumulated activity coefficients presented here should not be regarded as standardized values for dosimetric purposes, although the presented model may provide a useful tool for deriving them from a more extensive experimental setup.

The xenograft data was also well fitted by the proposed model. However, it should be taken into account that due to the lack of experimental data, especially for the blood and excretion compartments, the found combination of rate constants and blood fraction values might not be unique.

Compartmental biokinetic models are an important tool for reconstructing the time-activity curves of the radiopharmaceuticals used in molecular radiotherapy, and therefore to determine the absorbed doses. However, complex models present a great disadvantage towards individualized dosimetry in a real clinical context: the patient would have to undergo several measurement procedures comprising blood samples, urine collection, and imaging studies. In this regard, we have shown that a simplification of the model, although not so faithful in describing the biologic processes, allows reducing the number of required measurements without a great impact on the calculated cumulated activities. Therefore, it might be a better choice for practical dosimetric calculations. Simple phenomenological models, like exponential/biexponential fits, even if less accurate, may present more practical interest due to the limited data that they require. In general, they lead to less accurate fits, especially for some organs with particular biokinetics (see Figure 6 and Table 5 for the urinary bladder contents). Nonetheless, reconstructed cumulated activities with these simplified models are within [−13,24]% of values obtained with the full model for most organs/tissues, including the tumor.

Our analysis also provides some hints on the optimal imaging times to be able to obtain accurate cumulated activities with simple exponential (1 measurement) and biexponential models (4 measurements). In the case of exponential fits, it seems that performing the acquisition 2–3 days post-injection could provide in general more accurate cumulated activities (even though this requires further experimental validation). On the other hand, for the biexponential fit, better results are obtained when combining early (t≲1 days) and late (t≳4 days) acquisitions to account for *fast* and *slow* biokinetics, respectively.

The differences presented in Table 5 apply to the case of ^131^I. If the model was to be adapted for diagnostic dosimetric calculations with ^124^I, it is expected that the monoexponential fits would lead to even greater differences with the biokinetic model. This is a direct consequence of the shorter half-life of ^124^I, and therefore a greater contribution of the activity at early times to the total cumulated activity.

As far as we know, this is the first biokinetic model for the radiopharmaceutical ^124/131^I-CLR1404. This model may be helpful for individualized calculation of the activity distribution of the radiopharmaceuticals in a patient, providing that enough data is available. In addition, similarly to the biokinetic models of the ICRP [12], it might be employed in large studies to derive standardized cumulated activity coefficients for the dosimetry of ^124^I/^131^I-CLR1404.

## 5. Conclusions

Pharmacokinetic modeling of the radiopharmaceuticals used in molecular radiotherapy is an important step towards accurate radiation dosimetry of such therapies. We have developed a compartmental pharmacokinetic model for the ^124^I/^131^I-CLR1404, following a methodology that includes compartment decoupling, the use of a forcing function, Akaike Information Criterion, and sensitivity analysis. The model may prove useful to analyze further experimental data on the kinetics of this theragnostic agent. It may also assist in defining imaging protocols that allow the best possible reconstruction of time-activity curves. The developed compartmental structure and the methodology followed to construct the model should allow for a flexible re-definition and potential expansion if other experimental datasets become available.

## Figures and Tables

**Table 1:** Summary of the organ masses employed for scaling the activities per mass reported in [7] into total relative activities, and relative blood content of each organ: ^a^ Taken from Table A.2 of the ICRP-133 [31] (tissue with blood); ^b^ Taken from Table 2.8 of [30] (tissue plus blood contribution); ^c^ Mass reported by [7]; ^d^ Taken from Table 7.7.2 of [30].

Organ	Mass [g]	Initial Blood Fractions ^d^ [%]
Heart Wall	291 ^a^	1.0
Kidneys (both)	357 ^a^	2.0
Liver	1810 ^a^	10.0
Lungs (both)	950 ^b^	10.5
Bone Marrow	1064 ^a^	4.0
Spleen	187 ^a^	1.4
Tumor	38 ^c^	0.0

**Table 3:** Best-fitting parameter values and Monte Carlo calculated standard deviations obtained from 100 runs of the optimization algorithm with a dataset stochastically sampled from the experimental points and uncertainties.

Rate Constant [h^−1^]			
**Description**	**Value** ± **std**	**Description**	**Value** ± **std**
Blood to heart fast	$\left(2.3\pm 0.6\right)\times 10^{-1}$	Lung fast to slow	$\left(3.6\pm 1.0\right)\times 10^{-3}$
Heart fast to blood	(6.8±1.7)	Blood to liver fast	$\left(6.1\pm 3.1\right)\times 10^{-2}$
Heart fast to slow	$\left(9.3\pm 2.7\right)\times 10^{-4}$	Liver fast to blood	$\left(9.2\pm 3.7\right)\times 10^{-1}$
Blood to kidney fast	$\left(1.2\pm 0.7\right)\times 10^{-2}$	Liver fast to slow	$\left(2.5\pm 1.0\right)\times 10^{-3}$
Kidney fast to blood	$\left(4.1\pm 1.8\right)\times 10^{-1}$	Blood to BM fast	$\left(1.0\pm 0.3\right)\times 10^{-3}$
Kidney fast to slow	$\left(2.1\pm 0.7\right)\times 10^{-3}$	BM fast to blood	$\left(2.9\pm 1.3\right)\times 10^{-2}$
Blood to tumor fast	$\left(1.7\pm 0.6\right)\times 10^{-2}$	BM fast to slow	$\left(2.0\pm 0.9\right)\times 10^{-3}$
Tumor fast to blood	$\left(2.0\pm 0.8\right)\times 10^{1}$	Blood to RT fast	$\left(3.6\pm 0.4\right)\times 10^{-2}$
Tumor fast to slow	$\left(1.0\pm 0.2\right)\times 10^{-1}$	RT fast to blood	$\left(2.8\pm 0.4\right)\times 10^{-2}$
Tumor slow to fast	$\left(2.6\pm 0.5\right)\times 10^{-2}$	RT fast to RT slow	$\left(1.1\pm 0.2\right)\times 10^{-3}$
Blood to lung fast	$\left(3.4\pm 2.0\right)\times 10^{-3}$	Kidney fast to urine	$\left(1.9\pm 0.4\right)\times 10^{-2}$
Lung fast to blood	$\left(5.0\pm 3.1\right)\times 10^{-2}$	Blood to spleen	$\left(1.6\pm 0.3\right)\times 10^{-5}$
**Blood Fraction [%]**			
**Organ**	**Value** ± **std**	**Organ**	**Value** ± **std**
Bone marrow	3.9±0.2	Liver	8.2±1.5
Heart wall	0.8±0.1	Lungs	7.7±0.4
Kidneys	2.7±0.5	Spleen	1.3±0.1

**Table 4:** Example of some of the simplifications that were tested (with slow components removed for some organs), and their WRSS, goodness-of-fit, and Akaike Information Criterion values.

Description	WRSS	Goodness-of-Fit	${\mathrm{AIC}}_{c}$
Complete model	62.16	1.22	75.76
Heart slow compartment removed	76.92	1.48	87.93
Liver slow compartment removed	83.09	1.60	94.19
Liver and heart slow compartments removed	86.73	1.64	92.76
Slow compartments removed except for the tumor	108.90	1.91	93.40

**Table 5:** Relative differences in calculated cumulated activities of ^131^I-CLR1404 in several organs obtained with the *full* biokinetic model presented in this work ($\tilde{A}$) and several simplifications: “SM” (simplification of the full model without *slow* components except for RT and tumor); “BE1” (biexponential fit with the entire dataset); “BE2” (biexponential fit with only 4 points at [2, 24, 120, 505] h); “E 24 h”, “E 48 h”, “E 120 h” (exponential fits considering the physical decay of activities measured 24 h, 48 h, and 120 h post-injection, respectively).

$100\cdot (\tilde{A}$_simplification_−$\tilde{A}$)/$\tilde{A}$							
Organ	$\tilde{A}$ [%·h]	SM	BE1	BE2	E 24 h	E 48 h	E 120 h
Heart wall	482.60	−0.8%	0.5%	1.0%	23.0%	15.0%	−0.1%
Kidneys	648.14	−1.5%	−0.8%	2.5%	15.3%	3.6%	3.9%
Tumor	33.65	7.8%	6.3%	5.4%	6.8%	23.9%	11.1%
Lungs	1878.90	−3.3%	0.9%	−0.5%	−3.9%	−5.7%	−12.6%
Liver	1761.30	−1.3%	0.2%	1.1%	18.7%	6.3%	−3.6%
Bone marrow	824.43	−1.1%	−0.4%	−1.9%	1.5%	3.0%	−3.0%
Spleen	162.27	0.0%	0.0%	0.1%	5.6%	−1.0%	−5.3%
Bladder	1272.20	10.4%	173.3%	–	−74.6%	−59.2%	−46.2%

## Data Availability

Data and code supporting the reported results are available from the corresponding author.

## Supplementary Materials

The following are available at https://www.mdpi.com/article/10.3390/pharmaceutics13091497/s1, Table SM1. First-order sensitivity indexes Si, which account for the “relative importance” of the parameters at first order iterations. By definition, Si takes values between 0 and 1 and ΣSi≤1. The higher the Si value, the higher the relative importance of the parameter by firs-order iterations. A Si = 0 does not necessarilyimply null contribution to the model. Those indexes greater than zero were highlighted for a better visualization. Table SM2. Total-order sensitivity indexes ST,i, which account for the “relative importance” of the parameters to the model by higher order iterations, e.g., iterations of parameters with one another. By definition, ST,i ≥ Si. Figure SM1: Parallel coordinates plot representing the relative differences between the full compartmental model and bioexponential fits with different combinations of four time-points (Equation SM14). Figure SM2: Fitting of the complete model to the combined tissue activities reported for xenografts with tumor cell line CHLA20: (a) lungs, (b) heart, (c) kidneys, (d) liver, (e) marrow, (f) tumor. Figure SM3: Fitting of the complete model to the combined tissue activities reported for xenografts with tumor cell line RH30: (a) lungs, (b) heart, (c) kidneys, (d) liver, (e) marrow, (f) tumor. Figure SM4: Fitting of the complete model to the combined tissue activities reported for xenografts with tumor cell line NB1691: (a) lungs, (b) heart, (c) kidneys, (d) liver, (e) marrow, f) tumor. Figure SM5: Fitting of the complete model to the combined tissue activities reported for xenografts with tumor cell line TC71: (a) lungs, (b) heart, (c) kidneys, (d) liver, (e) marrow, (f) tumor.
