# Supplementary material for: Development of a Compartmental Pharmacokinetic Model for Molecular Radiotherapy with 131I-CLR1404
Source: Pharmaceutics. 2021 Sep 17;13(9):1497. doi: 10.3390/pharmaceutics13091497 (PMC8465706; doi:10.3390/pharmaceutics13091497)
Supplement: Supplementary file 1 [file pharmaceutics-13-01497-s001.zip › pharmaceutics-1327695-supplementary.pdf]

Supplementary Materials: Development of a compartmental pharmacokinetic model for molecular radiotherapy with ^{131}I-CLR1404

Sara Neira, Araceli Gago-Arias, Isabel González-Crespo, Jacobo Guiu-Souto, Juan Pardo-Montero

1. Differential equations of the model

Here we present the complete system of ODEs of the complete model, i.e. the expanded version of the Equation 4 in the article. In order to avoid extremely long expressions the following abbreviations were employed: LI = liver, BL = blood, LU = lungs, HT = heart, KD = kidneys, SP = spleen, BM = bone marrow, RT = remaining tissues, TM = tumor, UB = urinary bladder, TI = tissue, S = slow, F = fast. The differential equation for the blood compartment is given by:

$$\begin{aligned}\frac{dy_{BL}}{dt} =\ & k_{RT-F\rightarrow BL}\cdot y_{RT-F} + k_{LU-F\rightarrow BL}\cdot y_{LU} + k_{HT-F\rightarrow BL}\cdot y_{HT-F} + k_{KD-F\rightarrow BL}\cdot y_{KD-F} + k_{SP\rightarrow BL}\cdot y_{SP}\\ & + k_{BM-F\rightarrow BL}\cdot y_{BM-F} + k_{LI-F\rightarrow BL}\cdot y_{LI-F}\, k_{TM-F\rightarrow BL}\cdot y_{TM-F} - \left(\lambda + k_{BL\rightarrow RT-F} + k_{BL\rightarrow LU-F} + \right.\\ & \left. k_{BL\rightarrow HT-F} + k_{BL\rightarrow KD-F} + k_{BL\rightarrow SP} k_{BL\rightarrow BM-F} + k_{BL\rightarrow LI-F} + k_{BL\rightarrow TM-F}\right)\cdot y_{BL}\end{aligned} \qquad \text{(SM1)}$$

On the other hand, the fast (TI-F) and slow (TI-S) components for the compartments: lungs, heart wall, BM, remaining tissues and liver are described by:

$$\frac{dy_{TI-F}}{dt} = k_{BL\rightarrow TI-F}\cdot y_{BL} - \left(\lambda + k_{TI-F\rightarrow TI-S} + k_{TI-F\rightarrow BL}\right)\cdot y_{TI-F} \qquad \text{(SM2)}$$

$$\frac{dy_{TI-S}}{dt} = k_{TI-F\rightarrow TI-S}\cdot y_{TI-F} - \lambda\, y_{TI-S} \qquad \text{(SM3)}$$

and for the spleen we have:

$$\frac{dy_{SP}}{dt} = k_{BL\rightarrow SP}\, y_{BL} - \lambda\, y_{SP} \qquad \text{(SM4)}$$

In the special case of the tumor where the slow component does not behave like a sink, the activity evolves with time as:

$$\frac{dy_{TM-F}}{dt} = k_{BL\rightarrow TM-F}\cdot y_{BL} + k_{TM-S\rightarrow TM-F}\cdot y_{TM-S} - \left(\lambda + k_{TM-F\rightarrow TM-S} + k_{TM-F\rightarrow BL}\right)\cdot y_{TM-F} \qquad \text{(SM5)}$$

$$\frac{dy_{TM-S}}{dt} = k_{TM-F\rightarrow TM-S}\cdot y_{TM-F} - \left(\lambda + k_{TM-S\rightarrow TM-F}\right)\cdot y_{TM-S} \qquad \text{(SM6)}$$

Finally, for the kidneys and the urinary bladder we have the expressions:

$$\frac{dy_{KD-F}}{dt}=k_{BL\rightarrow KD-F}\cdot y_{BL}-(\lambda+k_{KD-F\rightarrow KD-S}+k_{KD-F\rightarrow BL}+k_{KD-F\rightarrow UB})\cdot y_{KD-F} \quad \text{(SM7)}$$

$$\frac{dy_{KD-S}}{dt}=k_{KD-F\rightarrow KD-S}\cdot y_{KD-F}-\lambda y_{KD-S} \quad \text{(SM8)}$$

$$\frac{dy_{UB}}{dt}=k_{KD-F\rightarrow UB}\cdot y_{KD-F}-\lambda y_{UB} \quad \text{(SM9)}$$

2. Sensitivity analysis

We relied on a sensitivity analysis based on conditioned variances (Saltelli et al. 2007, Saltelli et al 2010) to gain a better comprehension on the model behaviour under the perturbation of its parameters. The first- and total- order sensitivity indexes (S_i and $S_{T,i}$) were estimated for each model parameter in its first version (Figure 3 with dashed arrows), and the objective function WRSS, which was decoupled for each experimental organ data-series. Two sets of 31 x 9 (rate constants plus blood fractions x data series) for the S_i and $S_{T,i}$ indexes were obtained. The variance calculation was performed after a Monte Carlo computation, during which the model parameters were disturbed following a normal distribution of zero mean and relative standard deviation of 0.1. It involved a total computational cost of $N(K+2)$ runs, with $N=5\times10^4$ and K equal the number of model parameters. The main aim of this operation was to evaluate if there were room for model simplification ($S_{T,i}$ = 0) or indicatives for parameter prioritization (S_i ~ 1).

The relations that were employed in the indices calculation were those reported by Saltelli et al. (2011). Two matrices of N x K randomly perturbed parameters: A and B. Additionally a third matrix was defined as C_i equals A, except for the ith column which equals the ith column from B. From each parameter combination present in these matrices, the output from the model was obtained in the form of N x 1 vectors:

$$y_A=f(A) \qquad y_B=f(B) \qquad y_{C_i}=f(C_i) \quad \text{(SM10)}$$

Taking into account this notation, the Sobol indices were calculated from the relations:

$$V=\frac{1}{2N}\sum_{j=1}^{N}(y_{A,j}^2+y_{B,j}^2)-\left(\frac{1}{2N}\sum_{j=1}^{N}(y_{A,j}+y_{B,j})\right)^2 \quad \text{(SM11)}$$

$$S_i=\left[\frac{1}{N}\sum_{j=1}^{N}(y_{B,j}(y_{C_i,j}-y_{A,j}))\right]/V \quad \text{(SM12)}$$

$$S_{T,i}=\left[\frac{1}{2N}\sum_{j=1}^{N}\left(y_{A,j}-y_{C_i,j}\right)^2\right]/V \quad \text{(SM13)}$$

More details about these expressions can be found in Saltelli et al. (2010). The complete tables of S_i and $S_{T,i}$ coefficients are reported for each parameter and WRSS of each data-series, Tables SM1 and SM2.

3. Measurement time-points

As the patient was measured over several time points, we studied which was the optimum set of four acquisition times for fitting to a biexponential function. From the available data-set (8 time-points for the organs and tumor) we generated all the possible combinations for 4 points: a total of 70 possibilities. Finally, the estimated cumulated activity and the difference with that predicted by the compartmental model were calculated. The differences were defined as:

$$error=\frac{100}{N_{\text{organs}}}\sum_{\text{organs}}\frac{\left|\tilde{A}_{\text{biexp}}-\tilde{A}\right|}{\tilde{A}} \quad \text{(SM14)}$$

where $\tilde{A}$ is the cumulated activity predicted by the complete compartmental model and $\tilde{A}_{\text{biexp}}$ that obtained for the biexponential fit. Excretion data (urinary bladder activities) were excluded from this analysis, as it was shown in the article that biexponential fits cannot describe that compartment. The results are summarized in Figure SM1. The optimum scheme is reached by the measurements at [2h, 24h, 120h, 505h] for the organ and tumor.

$k_{i,j}$	Marrow	Heart wall	Kidneys	Liver	Lungs	Spleen	Tumor	Blood	Renal
Blood to heart fast	0.00	0.13	0.00	0.00	0.00	0.00	0.00	0.00	0.00
Heart fast to blood	0.00	0.25	0.00	0.00	0.00	0.00	0.00	0.00	0.00
Heart fast to slow	0.00	0.00	0.00	0.00	0.00	0.00	0.00	0.00	0.00
Blood to kidney fast	0.00	0.00	0.06	0.00	0.00	0.00	0.00	0.01	0.09
Kidney fast to blood	0.00	0.00	0.07	0.00	0.00	0.00	0.00	0.01	0.13
Kidney fast to slow	0.00	0.00	0.00	0.00	0.00	0.00	0.00	0.00	0.00
Blood to tumor fast	0.00	0.00	0.00	0.00	0.00	0.00	0.13	0.00	0.00
Tumor fast to blood	0.00	0.00	0.00	0.00	0.00	0.00	0.26	0.00	0.00
Tumor fast to slow	0.00	0.00	0.00	0.00	0.00	0.00	0.02	0.00	0.00
Tumor slow to fast	0.00	0.00	0.00	0.00	0.00	0.00	0.02	0.00	0.00
Blood to lung fast	0.00	0.00	0.00	0.00	0.07	0.00	0.00	0.00	0.00
Lung fast to blood	0.00	0.00	0.00	0.00	0.05	0.00	0.00	0.00	0.00
Lung fast to slow	0.00	0.00	0.00	0.00	0.01	0.00	0.00	0.00	0.00
Liver fast to blood	0.00	0.00	0.00	0.02	0.00	0.00	0.00	0.00	0.00
Blood to liver fast	0.00	0.00	0.00	0.01	0.00	0.00	0.00	0.00	0.00
Liver fast to slow	0.00	0.00	0.00	0.00	0.00	0.00	0.00	0.00	0.00
Blood to BM fast	0.05	0.00	0.00	0.00	0.00	0.00	0.00	0.00	0.00
BM fast to blood	0.05	0.00	0.00	0.00	0.00	0.00	0.00	0.00	0.00
BM fast to slow	0.00	0.00	0.00	0.00	0.00	0.00	0.00	0.00	0.00
Blood to RT fast	0.04	0.02	0.05	0.05	0.05	0.03	0.00	0.58	0.02
RT fast to blood	0.02	0.01	0.02	0.03	0.02	0.02	0.00	0.27	0.00
RT fast to RT slow	0.00	0.00	0.00	0.00	0.00	0.00	0.00	0.02	0.00
Kidney fast to urine	0.00	0.00	0.00	0.00	0.00	0.00	0.00	0.01	0.07
Spleen to blood	0.00	0.00	0.00	0.00	0.00	0.00	0.00	0.00	0.00
Blood to spleen	0.00	0.00	0.00	0.00	0.00	0.00	0.00	0.00	0.00
f_s									
f_{Blood}(BM)	0.18	0.00	0.00	0.00	0.00	0.00	0.00	0.00	0.00
f_{Blood}(Heart wall)	0.00	0.00	0.00	0.00	0.00	0.00	0.00	0.00	0.00
f_{Blood}(Kidneys)	0.00	0.00	0.06	0.00	0.00	0.00	0.00	0.00	0.00
f_{Blood}(Liver)	0.00	0.00	0.00	0.24	0.00	0.00	0.00	0.00	0.00
f_{Blood}(Lungs)	0.00	0.00	0.00	0.00	0.14	0.00	0.00	0.00	0.00
f_{Blood}(Spleen)	0.00	0.00	0.00	0.00	0.00	0.49	0.00	0.00	0.00

Table SM1. First-order sensitivity indexes S_i, which account for the "relative importance" of the parameters at first order iterations. By definition, S_i takes values between 0 and 1 and $\sum S_i \leq 1$. The higher the S_i value, the higher the relative importance of the parameter by firs-order iterations. A $S_i = 0$ does not necessarily imply null contribution to the model. Those indexes greater than zero were highlighted for a better visualization.

$k_{i,j}$	Marrow	Heart wall	Kidneys	Liver	Lungs	Spleen	Tumor	Blood	Renal
Blood to heart fast	0.00	0.54	0.00	0.00	0.00	0.00	0.00	0.00	0.00
Heart fast to blood	0.00	0.66	0.00	0.00	0.00	0.00	0.00	0.00	0.00
Heart fast to slow	0.00	0.02	0.00	0.00	0.00	0.00	0.00	0.00	0.00
Blood to kidney fast	0.00	0.00	0.45	0.00	0.00	0.00	0.00	0.01	0.48
Kidney fast to blood	0.00	0.00	0.42	0.00	0.00	0.00	0.00	0.01	0.53
Kidney fast to slow	0.00	0.00	0.05	0.00	0.00	0.00	0.00	0.00	0.00
Blood to tumor fast	0.00	0.00	0.00	0.00	0.00	0.00	0.52	0.00	0.00
Tumor fast to blood	0.00	0.00	0.00	0.00	0.00	0.00	0.66	0.00	0.00
Tumor fast to slow	0.00	0.00	0.00	0.00	0.00	0.00	0.21	0.00	0.00
Tumor slow to fast	0.00	0.00	0.00	0.00	0.00	0.00	0.12	0.00	0.00
Blood to lung fast	0.00	0.00	0.00	0.00	0.40	0.00	0.00	0.01	0.00
Lung fast to blood	0.00	0.00	0.00	0.00	0.32	0.00	0.00	0.00	0.00
Lung fast to slow	0.00	0.00	0.00	0.00	0.05	0.00	0.00	0.00	0.00
Liver fast to blood	0.00	0.00	0.00	0.20	0.00	0.00	0.00	0.00	0.00
Blood to liver fast	0.00	0.00	0.00	0.20	0.00	0.00	0.00	0.00	0.00
Liver fast to slow	0.00	0.00	0.00	0.04	0.00	0.00	0.00	0.00	0.00
Blood to red marrow fast	0.35	0.00	0.00	0.00	0.00	0.00	0.00	0.00	0.00
BM fast to blood	0.27	0.00	0.00	0.00	0.00	0.00	0.00	0.00	0.00
BM fast to slow	0.03	0.00	0.00	0.00	0.00	0.00	0.00	0.00	0.00
Blood to RT fast	0.34	0.20	0.34	0.35	0.34	0.30	0.08	0.64	0.15
RT fast to blood	0.22	0.12	0.23	0.23	0.22	0.18	0.04	0.32	0.09
RT fast to RT slow	0.01	0.01	0.01	0.01	0.01	0.01	0.00	0.03	0.00
Kidney fast to urine	0.00	0.00	0.01	0.00	0.00	0.00	0.00	0.01	0.45
Spleen to blood	0.00	0.00	0.00	0.00	0.00	0.00	0.00	0.00	0.00
Blood to spleen	0.00	0.00	0.00	0.00	0.00	0.05	0.00	0.00	0.00
f_s									
f_{Blood}(BM)	0.42	0.00	0.00	0.00	0.00	0.00	0.00	0.00	0.00
f_{Blood}(Heart wall)	0.00	0.06	0.00	0.00	0.00	0.00	0.00	0.00	0.00
f_{Blood}(Kidneys)	0.00	0.00	0.29	0.00	0.00	0.00	0.00	0.00	0.00
f_{Blood}(Liver)	0.00	0.00	0.00	0.60	0.00	0.00	0.00	0.00	0.00
f_{Blood}(Lungs)	0.00	0.00	0.00	0.00	0.34	0.00	0.00	0.00	0.00
f_{Blood}(Spleen)	0.00	0.00	0.00	0.00	0.00	0.85	0.00	0.00	0.00

Table SM2. Total-order sensitivity indexes $S_{T,i}$, which account for the “relative importance” of the parameters to the model by higher order iterations, e.g. iterations of parameters with one another. By definition, $S_{T,i} \geq S_i$.

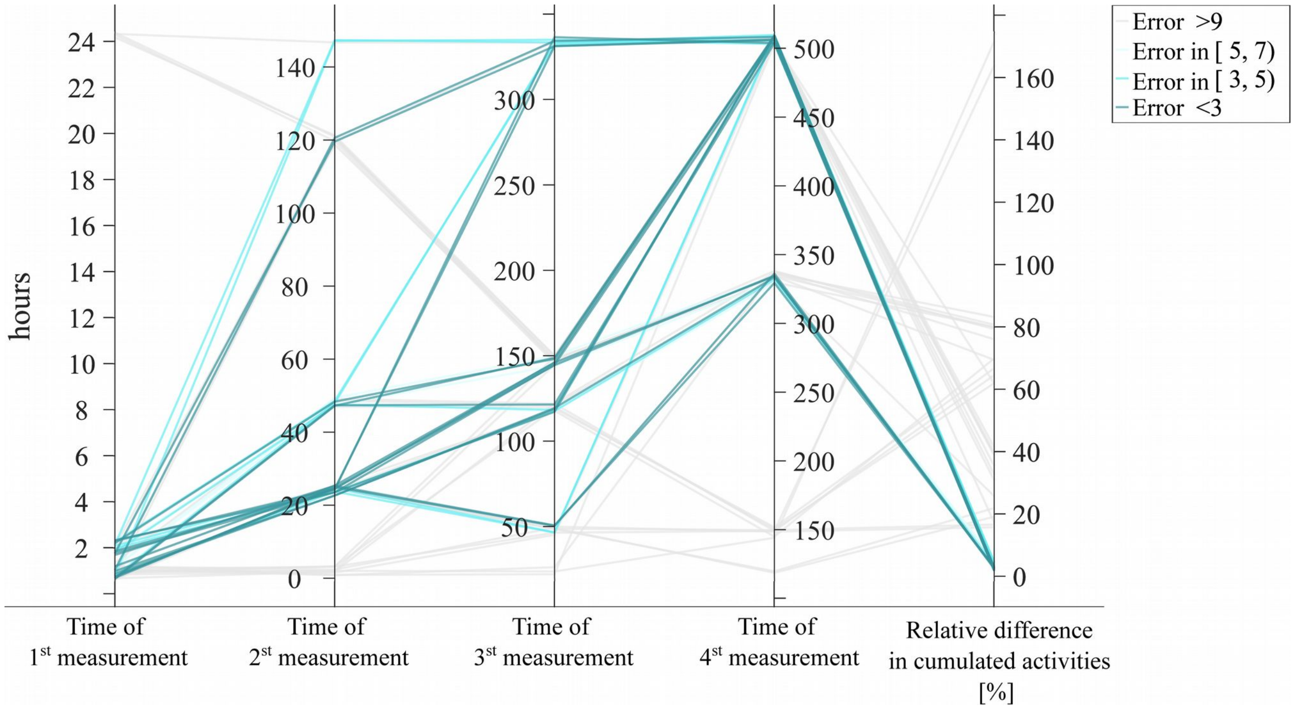

Figure SM1: Parallel coordinates plot representing the relative differences between the full compartmental model and bioexponential fits with different combinations of four time-points (Equation SM14).

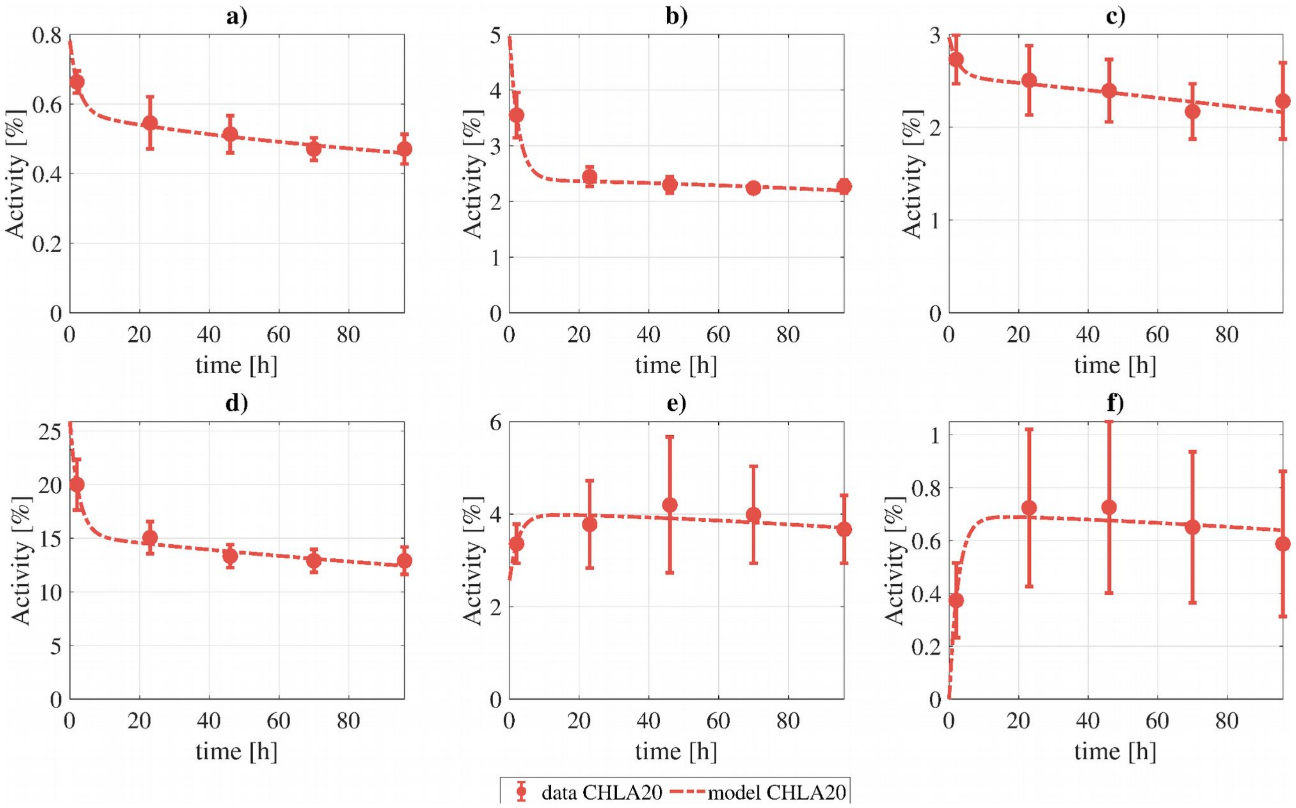

Figure SM2: Fitting of the complete model to the combined tissue activities reported for xenografts with tumor cell line CHLA20: a) lungs, b) heart, c) kidneys, d) liver, e) marrow, f) tumor.

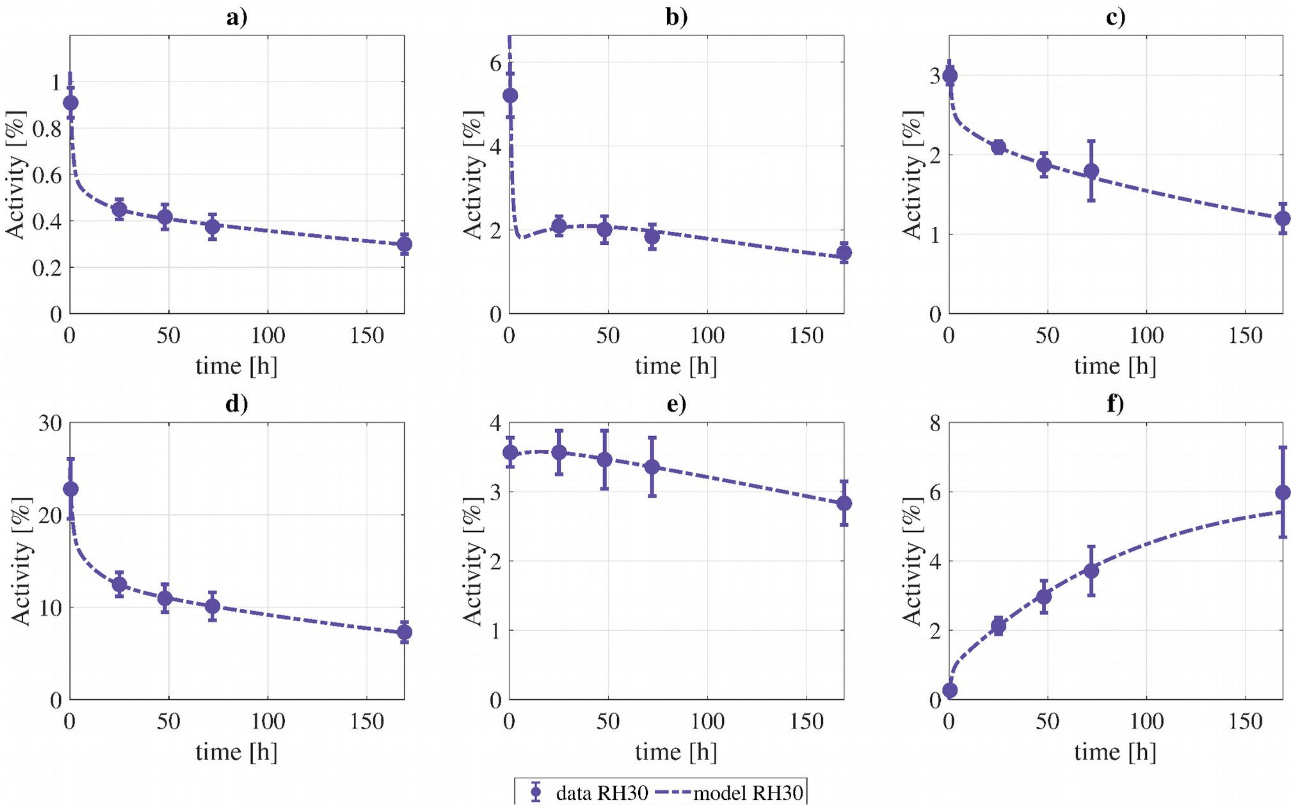

Figure SM3: Fitting of the complete model to the combined tissue activities reported for xenografts with tumor cell line RH30: a) lungs, b) heart, c) kidneys, d) liver, e) marrow, f) tumor.

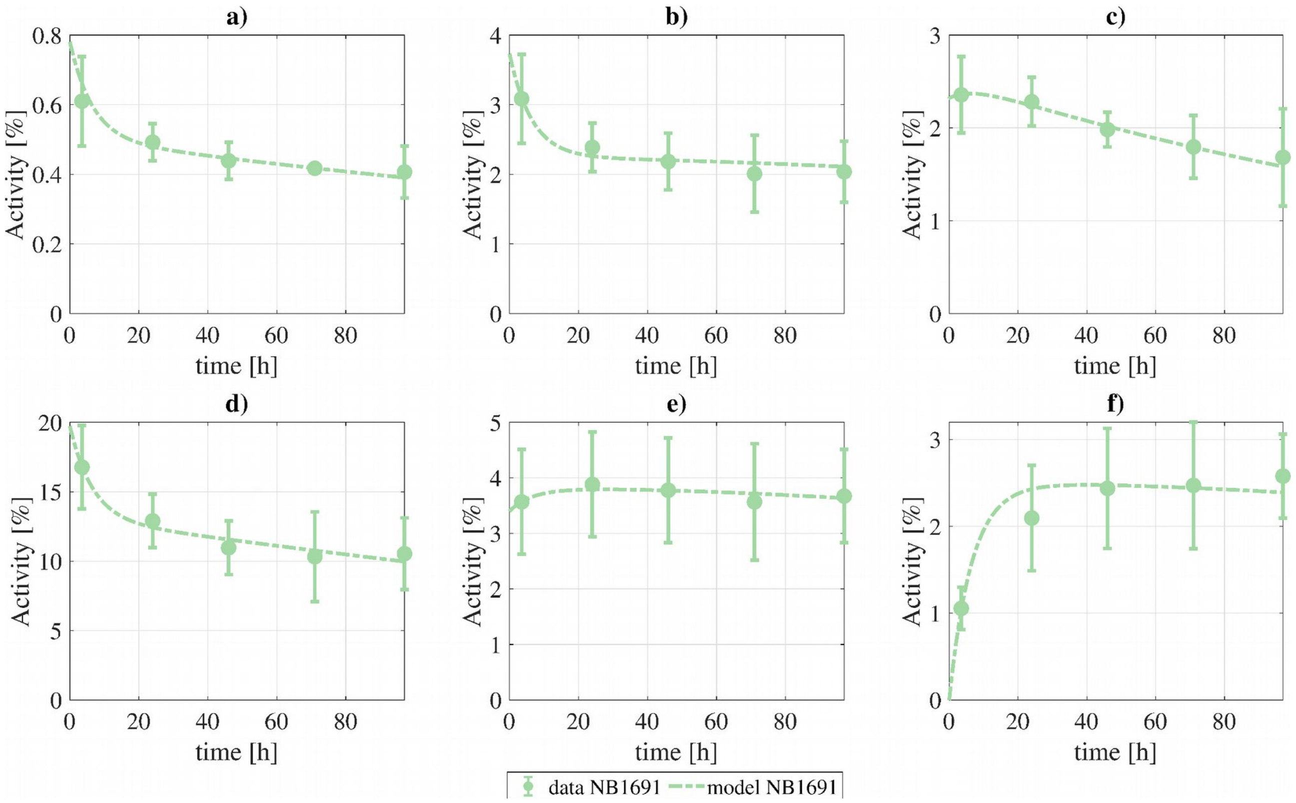

Figure SM4: Fitting of the complete model to the combined tissue activities reported for xenografts with tumor cell line NB1691: a) lungs, b) heart, c) kidneys, d) liver, e) marrow, f) tumor.

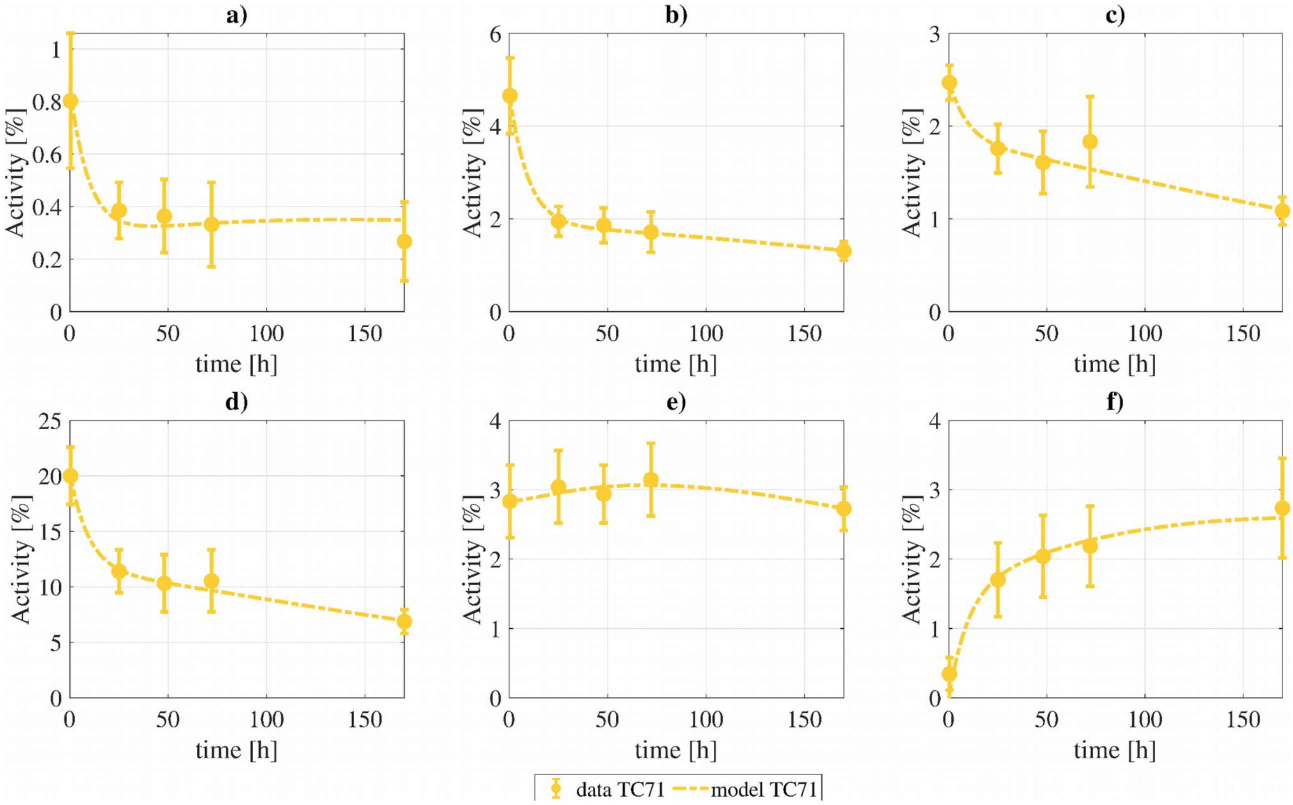

Figure SM5: Fitting of the complete model to the combined tissue activities reported for xenografts with tumor cell line TC71: a) lungs, b) heart, c) kidneys, d) liver, e) marrow, f) tumor.
